# Modulating the endoplasmic reticulum stress response attenuates neurodegeneration in a *Caenorhabditis* *elegans* model of spinal muscular atrophy

**DOI:** 10.1242/dmm.041350

**Published:** 2020-12-22

**Authors:** James J. Doyle, Celine Vrancx, Claudia Maios, Audrey Labarre, Shunmoogum A. Patten, J. Alex Parker

**Affiliations:** 1 Division of Experimental Medicine, McGill University, Montreal, Quebec H4A 3J1, Canada; 2 Metabolic Disorders and Complications, Research Institute of the McGill University Health Centre, Montreal, Quebec H4A 3J1, Canada; 3 Centre de Recherche du Centre Hospitalier de l'Université de Montréal and Department of Neuroscience, University of Montreal, Montreal, Quebec H2X 0A9, Canada; 4 INRS-Institut Armand-Frappier, Laval, Quebec H7V 1B7, Canada

**Keywords:** *Caenorhabditis elegans*, ER stress, Genetics, Muscle pathology, Spinal muscular atrophy

## Abstract

Spinal muscular atrophy (SMA) is a devastating autosomal recessive neuromuscular disease resulting in muscle atrophy and neurodegeneration, and is the leading genetic cause of infant death. SMA arises when there are homozygous deletion mutations in the human *SMN1* gene, leading to a decrease in corresponding SMN1 protein. Although SMN1 is expressed across multiple tissue types, much of the previous research into SMA focused on the neuronal aspect of the disease, overlooking many of the potential non-neuronal aspects of the disease. Therefore, we sought to address this gap in knowledge by modeling SMA in the nematode *Caenorhabditis elegans*. We mutated a previously uncharacterized allele, which resulted in the onset of mild SMA-like phenotypes, allowing us to monitor the onset of phenotypes at different stages. We observed that these mutant animals recapitulated many key features of the human disease, and most importantly, we observed that muscle dysfunction preceded neurodegeneration. Furthermore, we tested the therapeutic efficacy of targeting endoplasmic reticulum (ER) stress in non-neuronal cells and found it to be more effective than targeting ER stress in neuronal cells. We also found that the most potent therapeutic potential came from a combination of ER- and neuromuscular junction-targeted drugs. Together, our results suggest an important non-neuronal component of SMA pathology and highlight new considerations for therapeutic intervention.

## Abstract (precis)

**Summary:** A new non-larval-lethal *C. elegans* model of spinal muscular atrophy shows mild phenotypes, such as muscle cell and neuronal degeneration, and is therefore useful for testing potential drug treatments.

## INTRODUCTION

Spinal muscular atrophy (SMA) is an autosomal recessive neuromuscular disorder of early childhood. The incidence of SMA is ∼1 in 10,000 live births, and it is reported to be the leading genetic cause of infant death (Prior, 2008; Sugarman et al., 2012). SMA is characterized by the progressive loss of motor neurons in the anterior horn of the spinal cord (Moultrie et al., 2016). This degeneration leads to muscle weakness and atrophy, primarily affecting proximal muscles and lung muscles, resulting in limb paralysis, respiratory failure and death. SMA can manifest itself in several degrees of severity but all have in common progressive muscle atrophy and mobility impairment.

In humans, there are two copies of the survival motor neuron gene, *SMN1* and *SMN2*. The *SMN2* gene is almost identical to the *SMN1* gene, with the difference being that it holds a C-to-T nucleotide exchange at position 6 of exon 7, which is responsible for the skipping of exon 7 during splicing (Jablonka and Sendtner, 2017). This alternative splicing causes 90% of transcripts to result in a truncated protein, SMNΔ7, which degrades rapidly. The other 10% undergo correct splicing, thus producing a small amount of SMN protein. In healthy individuals, the *SMN1* gene can compensate for overall SMN protein production; however, in more than 90% of SMA cases, there is a homozygous deletion or mutation in the *SMN1* (Lefebvre et al., 1995; Farrar and Kiernan, 2015) gene, resulting in an inability of *SMN1* to counterbalance SMN protein levels. Under normal circumstances, SMN1 is involved in many aspects of RNA processing in cells, notably in the splicing of pre-mRNA to mRNA (Peeters et al., 2014). It is also required for proper neuronal outgrowth to form axons and dendrites.

As *SMN1* and *SMN2* are evolutionarily conserved, we used the nematode *Caenorhabditis elegans* to develop a new biologically relevant animal model to study SMA pathology (Owen et al., 2000). Although nematodes and humans are evolutionarily distant, genetically they share a high degree of conservation, and *C. elegans* has been widely used to model many aspects of neurodegeneration (Duronio et al., 2017). Both human *SMN1* and *SMN2* genes have a single ortholog in nematodes, named *smn-1* (Owen et al., 2000). Previous studies into *C. elegans smn-1* have primarily used the *ok355* deletion mutant, which, as in humans, results in lethality (Dimitriadi et al., 2016, 2010, 2013; Briese et al., 2009; Gao et al., 2014; O'Hern et al., 2017; Riessland et al., 2017). Although it recapitulates the aspects of the human disease, it is impractical to study finer aspects of the disease, such as phenotype and symptom onset. For this, we sought to study *smn-1* mutations using a mild mutant allele. Using the *gk118916* allele, which has a C-to-T nucleotide exchange in exon 4 of *smn-1*, we show that animals with this mild mutation still exhibit key features of the human disease, including reduced lifespan, progressive age-dependent paralysis and neurodegeneration, but do not show a strong larval-lethal phenotype. Furthermore, a key feature apparent in SMA is the presence of ER stress (Ng et al., 2015), which arises from the abnormal splicing of ER chaperones. This feature is also conserved in our nematode model, and is a potential therapeutic target.

Although much of the previous research into SMA has focused on the role of SMN1 in neurons and on the neuronal component of the pathology of SMA, SMN1 is expressed in many tissues and cell types. Therefore, it may be informative to investigate SMN1 and SMA pathology beyond their neuronal components (Nash et al., 2016). In this study, we used the *smn-1(gk118916)* allele [herein referred to as *smn-1(gk)*] to generate a moderate SMA model. The mild model has many advantages over the severe lethal alleles, notably that it is possible to study the temporal onset of several phenotypes in the model. We discovered that, surprisingly, muscle degeneration preceded neurodegeneration, and that genetically inhibiting ER stress in non-neuronal cells alone suppressed degenerative phenotypes. We further show that a combination of small molecules targeting both ER stress and neuromuscular junctions (NMJs) is more effective than the individual treatments. Together, our results introduce a new genetically relevant model to study SMA, which can be used for SMA drug discovery and development.

## RESULTS

### An *smn-1* point mutation leads to motor defects and neurodegeneration in *C. elegans*

**Fig. 1.**
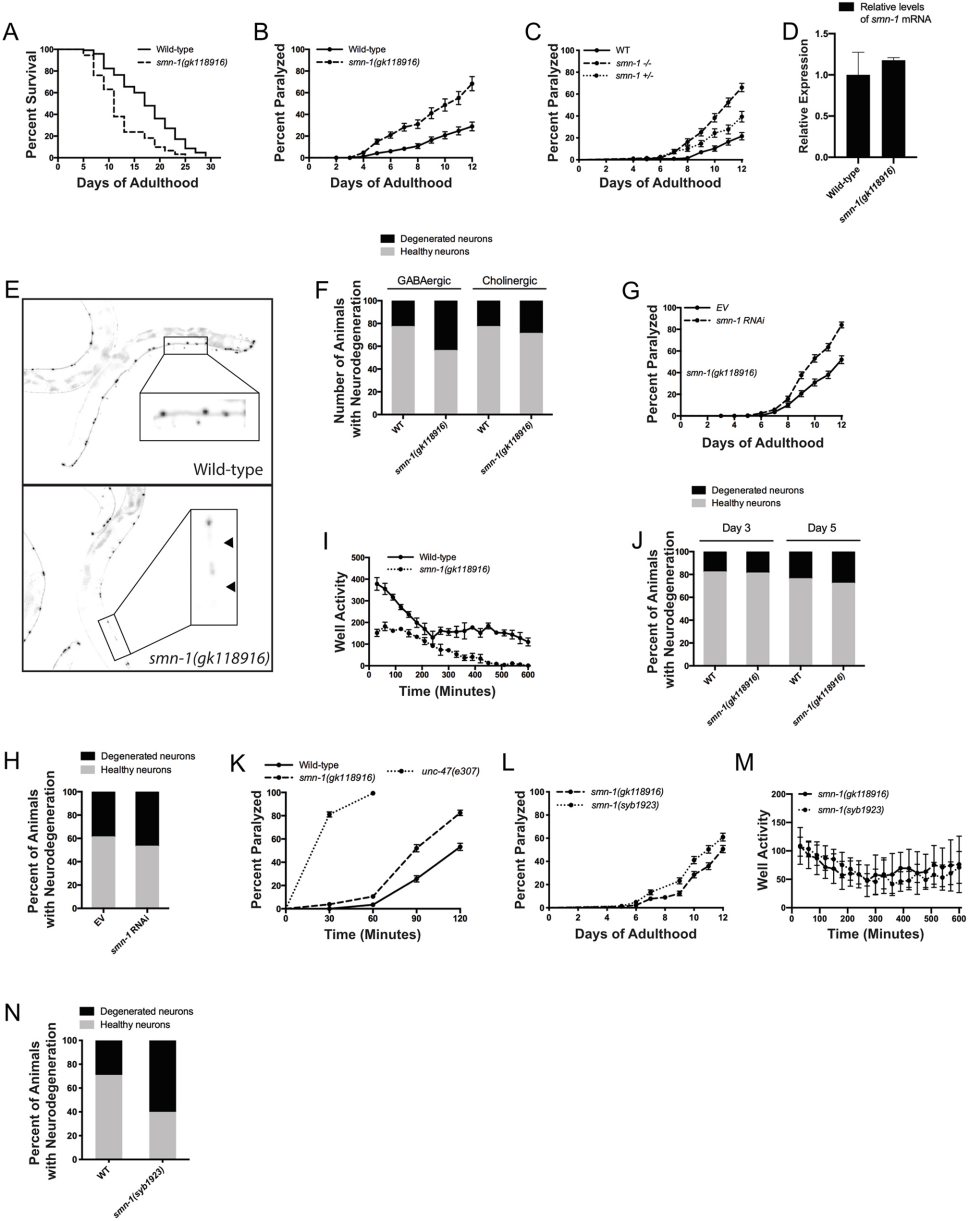
**A point mutation in *smn-1* recapitulates key neuronal features of SMA.** (A) *Smn-1(gk)* mutant animals display a reduction in lifespan (*P*<0.0001). (B) *Smn-1(gk)* mutants exhibit an increase in age-dependent paralysis compared to wild-type N2 animals (*P*<0.0001). (C) *smn-1(gk)*/^+^ heterozygous animals display lower paralysis levels compared to *smn-1(gk)*/ *smn-1(gk)* homozygous mutants, but higher paralysis than N2 animals (Mantel–Cox test, *P*<0.0001 and *P*<0.001, respectively). (D) The *smn-1(gk)* mutation does not significantly affect *smn-1* mRNA expression levels. (E) Representative images of GABAergic neurodegeneration observed in day 9 adult wild-type and *smn-1(gk)* mutant animals. Black arrowheads indicate neuronal gaps indicative of neurodegeneration. (F) *Smn-1(gk)* mutant animals display GABAergic motor neuron degeneration at day 9 (*P*<0.001, *n*=100 animals per condition), but do not show any degeneration of cholinergic motor neurons (*n*=100 animals per condition). (G) Non-neuronal RNAi treatment of *smn-1(gk)* animals with *smn-1* RNAi exacerbates paralysis (*P*<0.0001). (H) Non-neuronal *smn-1* RNAi treatment does not increase GABAergic motor neuron degeneration in *smn-1(gk)* mutants (*n*=100 animals per condition). (I) Swimming defects in *smn-1(gk)* animals are observed at day 5 of adulthood (*P*<0.0001). (J) *Smn-1(gk)* mutant animals do not display degeneration of GABAergic motor neurons at days 3 or 5 (not significant, *n*=100 animals per condition). (K) At day 1 of adulthood, *smn-1(gk)* mutants display hypersensitivity to aldicarb compared to N2 animals, but not to the same extent as *unc-47(e307)* animals (*P*<0.0001 and *P*<0.0001, respectively). (L) *Smn-1(syb1923)* animals show slightly higher levels of paralysis than *smn-1(gk)* animals (*P*<0.01). (M) Swimming defects in *smn-1(syb1923)* are nearly identical to those in *smn-1(gk)* mutants. (N) *Smn-1(syb1923)* animals display GABAergic neuron degeneration (*P*<0.01, *n*=100 animals per condition). Data are mean±s.d. Statistical significance was determined using a Mantel–Cox test (A-C,G,K,L), two-tailed unpaired Student's *t*-test (D,F,H,J), or two-way ANOVA (I,M).

The C-to-T nucleotide change in the chosen *smn-1* allele, *gk118916*, results in an E167K missense mutation at the protein level and was generated by the Million Mutation Project (MMP) (Thompson et al., 2013). Although not a splice-site mutation as found in humans, the amino acid change from a negatively charged glutamine (E) residue to a positively-charged lysine (K) is predicted to impair the protein structure of SMN-1. *Smn-1(gk)* mutant animals do not display any gross developmental problems and appear superficially normal, but upon closer inspection of adult worms we observed a reduction in lifespan (Fig. 1A). The normal development into adulthood helps remove the constraints on studying SMA in *C. elegans* as this viable allele allows for a wider range of analyses. We also observed motility defects in these animals, leading to age-dependent paralysis a few days after the worms entered adulthood (Fig. 1B).

As SMA is caused by mutations resulting in decreased SMN protein levels, we hypothesized that the paralysis phenotype observed in *smn-1(gk)* mutants was due to an insufficient level of wild-type SMN-1 protein. We investigated this possibility by crossing the *smn-1(gk)* strain to wild-type N2 animals to create *smn-1(gk)*/^+^ animals, and we observed that the level of paralysis for these heterozygotes was less than *smn-1(gk)*/*smn-1(gk)* mutants, but greater than wild-type +/+ N2 worms (Fig. 1C). Lacking a functional antibody targeting the endogenous SMN-1 protein, we looked at gene expression levels of *smn-1* mRNA and saw a slight non-significant increase in *smn-1* transcript levels in *smn-1(gk)* animals compared to N2 controls (Fig. 1D). Although not significant, this increase in mRNA levels could be explained by a feedback mechanism seeking to compensate for a loss of the normal function of SMN-1 protein. Together, these data suggest that *smn-1(gk)* acts in a semi-dominant manner, and the paralysis phenotype may correspond to a partial loss of SMN-1 protein function, as opposed to a decrease in the total amount of SMN-1 protein.

Motility problems can be a sign of neuronal dysfunction and/or neurodegeneration, as we have previously observed in our worm models of amyotrophic lateral sclerosis (ALS) (Vaccaro et al., 2012). To investigate the possibility of motor neuron degeneration, we crossed the *smn-1(gk)* mutants with transgenic reporter strains expressing fluorescent proteins in GABAergic (Barbagallo et al., 2010) or cholinergic motor neurons. Degeneration of motor neurons was assessed by scoring for the presence of breaks, or gaps, along the ventral nerve cord (Fig. 1E). Compared to wild-type controls, at day 9 of adulthood we observed a significant increase in the degeneration of GABAergic motor neurons, but no significant difference was noted for cholinergic motor neurons (Fig. 1F).

As human *SMN1* mutations that lead to SMA are a complete loss of function (LOF), we sought to better understand the nature of the mutations in the *smn-1(gk)* nematodes. Therefore, we used RNA interference (RNAi) to further reduce the amount of *smn-1* available, and observed a significant increase in paralysis in *smn-1(gk)-smn-1(RNAi)* animals, but did not see a further enhancement of neurodegeneration (Fig. 1G,H). These data suggest that *smn-1(gk)* is a hypomorphic allele resulting in a partial LOF.

We sought to further identify phenotypes characteristic of these *smn-1(gk)* nematodes, so we assessed their ability to swim. When nematodes are placed in a liquid medium such as M9 they change their characteristic crawling movement for a thrashing swimming-like motion, which can be analyzed over a period of time. We assayed for swimming at both day 1 and day 5 of adulthood, and observed that *smn-1* mutants did not display any difficulty swimming at day 1 (data not shown), but there was a clear impairment of swimming at day 5 (Fig. 1I) compared to N2 control animals. The onset of impaired swimming defects correlated with the onset of the paralysis phenotypes for *smn-1(gk)* worms grown on solid medium. As there was no observable neurodegeneration at earlier stages (days 3 and 5, Fig. 1J), we sought to identify the causes of the swimming and paralysis phenotypes. We have shown in previous work that neurotransmission is affected in *C. elegans* models of ALS, and as ALS and SMA are very similar clinically (Bowerman et al., 2017), we tested synaptic function in *smn-1(gk)* animals. Upon treatment with aldicarb, an acetylcholinesterase inhibitor (Mahoney et al., 2006), *smn-1* mutant worms displayed a hypersensitivity and became paralyzed sooner than control animals, but not to the extent of *unc-47(e307)* positive control animals (Fig. 1K). This suggested that our *smn-1(gk)* animals may have NMJ defects.

A common criticism of strains generated by the MMP is that they contain on average ∼400 mutations. As a result, even after six rounds of outcrossing, there is a risk that any phenotype potentially linked to a mutation of interest may be due to another mutation that remained in the strain background. Therefore, we used CRISPR/Cas9 (Paix et al., 2014, 2015, 2017; Dickinson and Goldstein, 2016) to introduce the *gk118916* allele into wild-type N2 animals to see if they would exhibit similar phenotypes. The CRISPR mutants, *smn-1(syb1923)*, exhibited slightly higher levels of paralysis compared to *smn-1(gk)* animals but had a nearly identical swimming defect (Fig. 1L,M). These CRISPR animals also displayed significant levels of neurodegeneration in their GABAergic neurons (Fig. 1N). Therefore, we can conclude that phenotypes observed in *smn-1(gk)* animals can be attributed to the *gk118916* allele and not to a background mutation still present in the strain. Together, these data show that *smn-1(gk)* may be a suitable and relevant model to study SMA as it recapitulates many key features of the disease. Last, as we observed that motility defects can be detected in the absence of neurodegeneration, there may be additional factors contributing to impaired movement of *smn-1* mutants.

### *smn-1(gk)* mutations lead to loss of muscle cell integrity

**Fig. 2.**
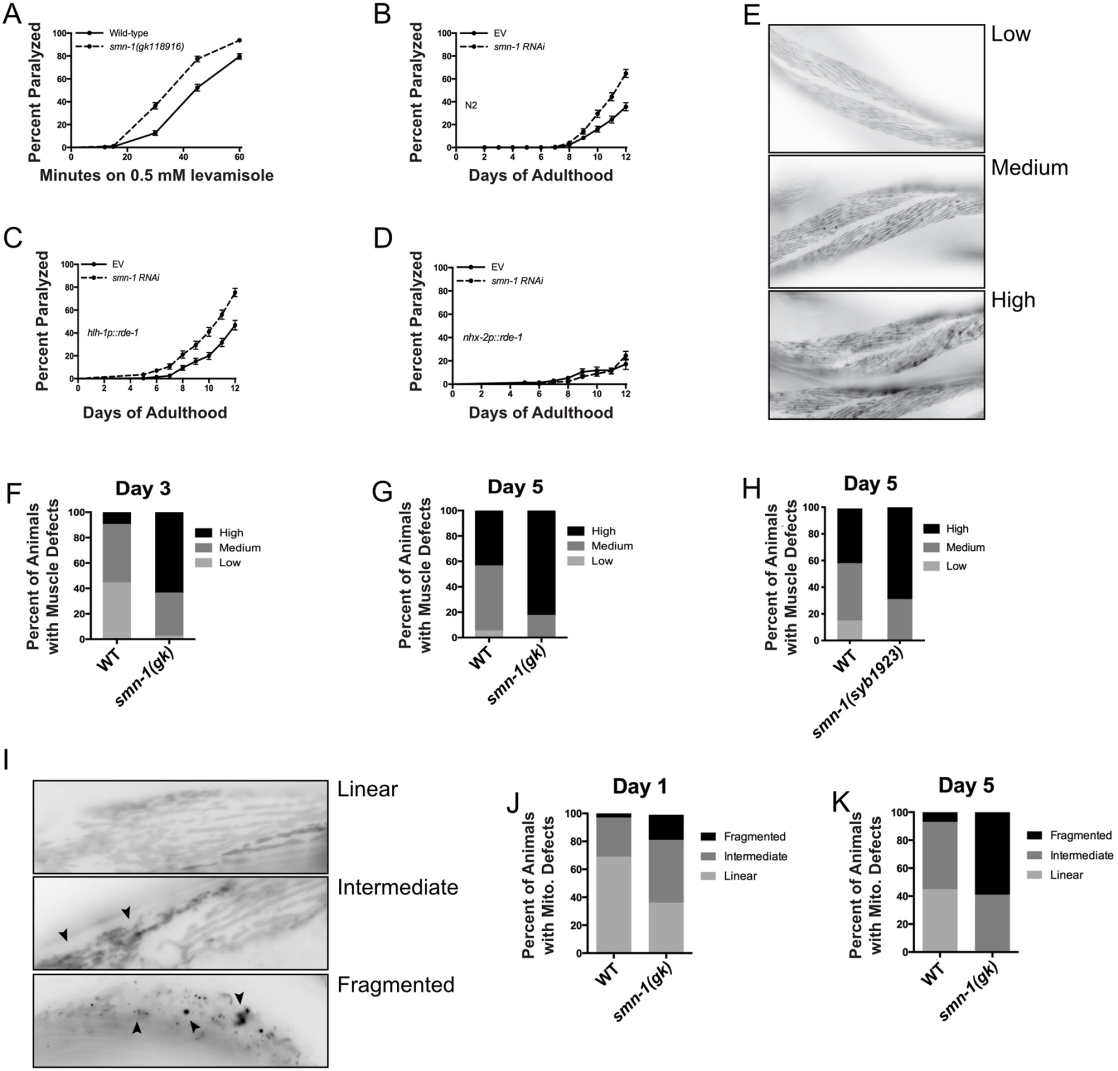
***smn-1(gk)* mutants show signs of early muscle defects.** (A) Day 1 adult *smn-1(gk)* mutant animals are hypersensitive to the nAChR agonist levamisole (*P*<0.0001). (B) Wild-type N2 animals become paralyzed when they are fed RNAi against *smn-1* (*P*<0.0001). (C) Transgenic worms sensitive to RNAi only in their body-wall muscle cells became paralyzed when they were fed RNAi against *smn-1* (*P*<0.0001). (D) RNAi knockdown of *smn-1* in intestinal cells did not result in a paralysis phenotype. (E) Representative images of transgenic animals expressing GFP::MYO-3 in their body-wall muscle cells. Levels of morphological distortion were characterized as ‘low’, ‘medium’, or ‘high’ depending on the extent of GFP::MYO-3 disorganization in the cells. (F,G) At days 3 and 5 of adulthood, greater numbers of *smn-1(gk)* mutants displayed ‘high’ levels of muscle dysfunction (*P*<0.0001, *n*=100 animals per condition). (H) Smn-1(syb1923) CRISPR mutants displayed increased muscle dysfunction compared to wild-type animals (*P*<0.0001). (I) Representative images of transgenic animals expressing TOM20::mRFP in body-wall muscle cells. Mitochondrial organization was quantified as either ‘linear’, ‘intermediate’, or ‘fragmented’. Arrowheads indicate muscle defects. (J,K) At days 1 and 5, *smn-1(gk)* mutants had higher levels of mitochondria impairment compared to wild-type animals (*P*<0.0001, *n*=100 animals per condition). Data are mean±s.d. Statistical significance was determined using a Mantel–Cox test (A-D) or two-way ANOVA (F-H,J,K).

It is widely accepted that SMA is a neuromuscular disorder; however, we wanted to investigate whether the *smn-1(gk)* mutants had any non-neuronal consequences. We tested *smn-1(gk)* animals for their sensitivity to levamisole, a nicotinic acetylcholine receptor agonist (nAChR) (Miller et al., 1996). As nAChRs are solely expressed in body-wall muscle cells, this assay was used to identify potential defects in the muscle cells of the worms. We observed that day 1 adult *smn-1(gk)* animals were hypersensitive to levamisole, suggesting the presence of very early muscle defects (Fig. 2A). Therefore, we performed the non-neuronal RNAi knockdown of *smn-1* in wild-type N2 animals and in a transgenic strain sensitive to RNAi, specifically in body-wall muscle cells (Fig. 2B,C). It was evident that RNAi-mediated depletion of *smn-1* was sufficient to induce paralysis phenotypes in these two strains, but the depletion of *smn-1* only in intestinal cells had no effect on animal health (Fig. 2D). These data suggest there may be a contribution of SMN-1 in muscle cells to worm health and viability.

We then sought to investigate whether there was any evidence of muscle morphological defects in *smn-1(gk)* animals. We used a recombinant myosin protein tagged with GFP, MYO-3::GFP (Meissner et al., 2009), to visualize muscle integrity. Under normal circumstances, the MYO-3::GFP signal can be observed as regular linear filaments within the body-wall muscles, as would be expected from myosin filaments (Meissner et al., 2009; Moerman and Fire, 1997). However, we observed, with age, the appearance of irregular shapes or disorganized GFP, suggesting a loss of myosin structural integrity, and a likely loss of general muscle structure. We characterized the extent of this disorganization as ‘low’, ‘medium’ or ‘high’ (Fig. 2E), which allowed us to quantify this morphological defect. As early as day 3 of adulthood, we observed a significant increase of *smn-1(gk)* animals with high levels of disorganization compared to wild-type animals (Fig. 2F), and this effect was worsened in day 5 animals (Fig. 2G). This phenotype was also present in *smn-1(syb1923)* CRISPR mutants at day 5 (Fig. 2H). Similarly, we quantified levels of mitochondrial fragmentation in body-wall muscle cells using a TOM20::mRFP reporter as a complementary assay of muscle health. We characterized mitochondrial organization as ‘linear’, ‘intermediate’ or ‘fragmented’ (Fig. 2I), as described previously (Sarasija and Norman, 2015, 2018). We observed that as early as day 1, we see a significant increase in levels of ‘fragmented’ mitochondria (Fig. 2J), with this effect being significantly aggravated by day 5 (Fig. 2K). In light of our observations about the lack of neurodegeneration at days 3 to 5 in adult *smn-1* mutants (Fig. 1I), our data suggest that in the absence of wild-type levels of SMN-1 protein, muscle cell defects occur before motor neuron degeneration, a phenomenon that may have implications in the design of therapeutic approaches for SMA.

### Small molecule regulators of ER stress rescue SMA-like phenotypes in *C. elegans*

**Fig. 3.**
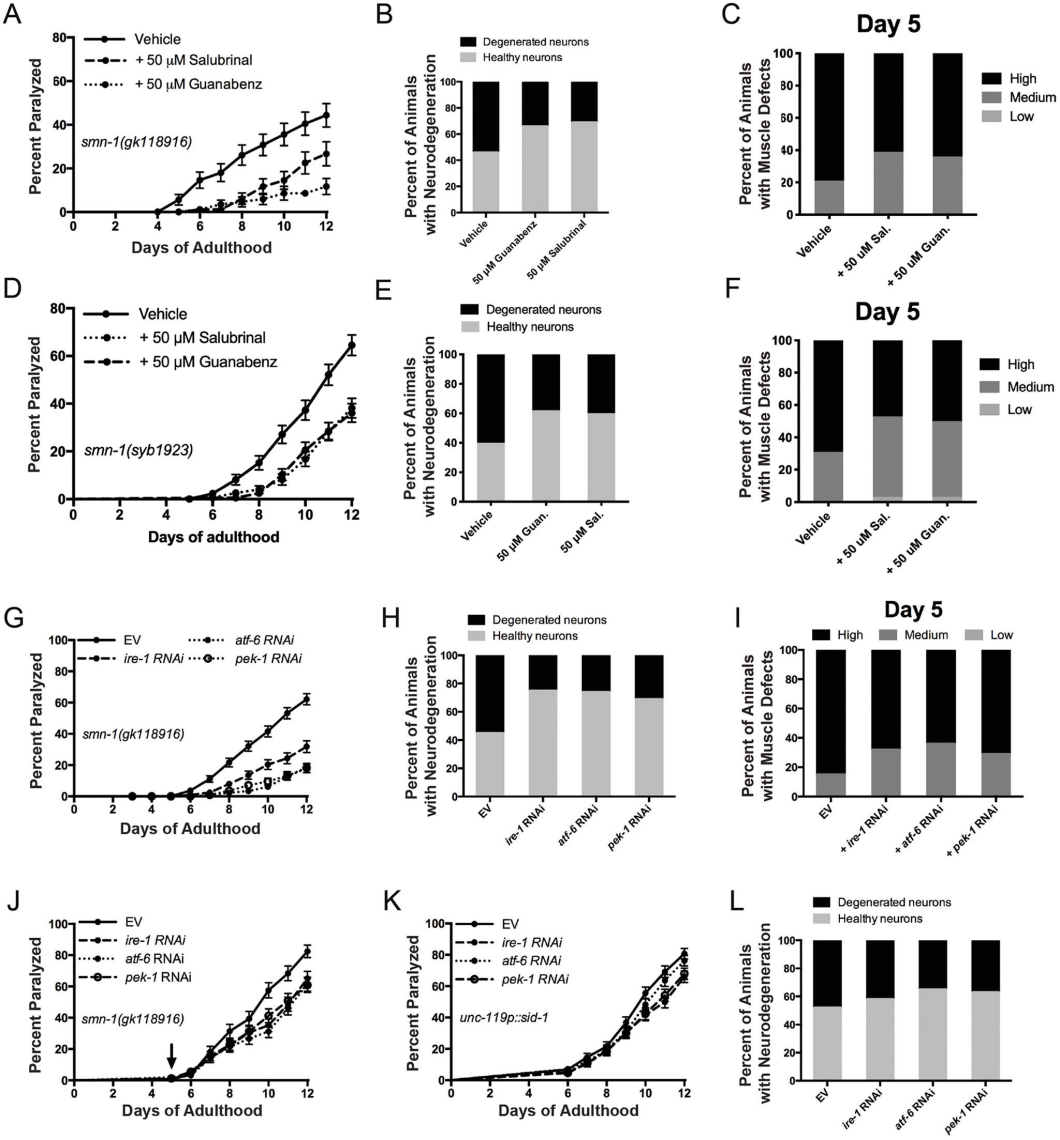
**Targeting ER stress chemically and genetically is protective in *smn-1* mutants, and is more effective when targeted to non-neuronal cells.** (A) Treatment of *smn-1(gk)* mutant animals with either 50 µM guanabenz or salubrinal attenuates the paralysis phenotype (*P*<0.0001 and *P*<0.01, respectively). (B) Pharmacological inhibition of ER stress with either 50 µM guanabenz or salubrinal prevents neurodegeneration in GABAergic motor neurons in *smn-1(gk)* mutants (*P*<0.05 and *P*<0.01, respectively; *n*=100 animals per condition). (C) Salubrinal and guanabenz treatment partially restores muscle morphology in *smn-1(gk)* mutants (*P*<0.0001 and *P*<0.0001, respectively). (D) Treating *smn-1(syb1923)* animals with either 50 µM guanabenz or salubrinal ameliorates their paralysis phenotype (*P*<0.0001). (E) Salubrinal and guanabenz treatments restore neurodegeneration at day 9 in *smn-1(syb1923)* animals (*P*<0.001; *n*=100 animals per condition). (F) Salubrinal and guanabenz treatments partially restore muscle dysfunction of *smn-1(syb1923)* mutants (*P*<0.01 and *P*<0.05, respectively). (G) Non-neuronal RNAi against *ire-1*, *atf-6* and *pek-1* restores paralysis in *smn-1(gk)* animals compared to EV treatment (*P*<0.0001). (H) Treatment of *smn-1(gk)* animals with non-neuronal *ire-1*, *atf-6* and *pek-1* RNAi also protects against neurodegeneration (*P*<0.0001, *P*<0.001 and *P*<0.01, respectively; *n*=100 animals per condition). (I) Treating *smn-1(gk)* mutants with non-neuronal *ire-1*, *atf-6* and *pek-1* RNAi helps restore muscle defects (*P*<0.0001, *n*=100 animals per condition). (J) Non-neuronal *ire-1*, *atf-6* and *pek-1* RNAi treatment from day 5 of adulthood is still capable of partially attenuating paralysis phenotypes in *smn-1(gk)* mutants (*P*<0.01, *P*<0.001 and *P*<0.01, respectively). (K) Neuronal *ire-1*, *atf-6* and *pek-1* RNAi treatment had no effect on *smn-1(gk)* paralysis compared to EV treatment. (L) Neuronal knockdown of *ire-1*, *atf-6* *pek-1* in *smn-1(gk)* animals does not significantly rescue neurodegeneration (*n*=100 animals per condition). Levels of morphological distortion were characterized as ‘low', ‘medium' or ‘high’ depending on the extent of GFP::MYO-3 disorganization in the cells. Data are mean±s.d. Statistical significance was determined using a Mantel–Cox test (A,D,G,J,K), a two-tailed unpaired Student's *t*-test (B,E,H,L) and two-way ANOVA (C,F,I).

Previously, our group has studied the role of ER stress in ALS models, and we have identified compounds able to correct motor neuron phenotypes in *C. elegans* and zebrafish (Vaccaro et al., 2013). As ALS and SMA share many clinical and molecular features, we tested whether two ER stress-targeting compounds, guanabenz and salubrinal, had protective effects in our *smn-1(gk)* model. We observed that treatment with either compound rescued paralysis and neurodegeneration phenotypes in mutant *smn-1* animals (Fig. 3A,B). Furthermore, when worms were treated with the compounds, from the L4 larval stage until day 5 of adulthood, there was a marked reduction in muscle defects apparent in the mutant animals (Fig. 3C). The protective effects of both compounds were maintained in *smn-1(syb1923)* mutants against their paralysis, neurodegeneration and muscle defects (Fig. 3D-F).

### Targeting ER stress in non-neuronal cells alleviates motor neuron degeneration

As muscle defects appear earlier than neuronal problems in *smn-1* mutants, we next wanted to investigate whether targeting ER stress in non-neuronal cells would have a stronger beneficial effect than in neuronal cells. In *C. elegans*, neuronal cells are naturally resistant to RNAi knockdown and must be genetically modified to be sensitive to RNAi in neurons. Therefore, when non-transgenic animals are treated with RNAi, the knockdown effect is mainly experienced in non-neuronal cells. We used RNAi against *ire-1*, *atf-6*, and *pek-1*, three upstream effectors of ER stress and nematode orthologs of human IRE1 (also known as ERN1), ATF6 and PERK (also known as EIF2AK3) (Vaccaro et al., 2013; Richardson et al., 2011), respectively, and an empty vector (EV) control. When *smn-1* mutants were treated with non-neuronal ER RNAi from the L4 stage, we observed a significant decrease in progressive paralysis and neurodegeneration in day 9 animals, and muscle defects in day 5 adult animals (Fig. 3G-I). These results were unexpected and suggest that genetically blocking ER stress in non-neuronal cells can reduce the degeneration of motor neurons.

To check whether the age-related phenotypes caused by *smn-1(gk)* mutations could be reversed at a later stage, we treated mutant animals with non-neuronal RNAi against *ire-1*, *atf-6* and *pek-1* commencing at day 5 of adulthood. We saw a significant decrease in paralysis levels in these animals compared to the EV controls (Fig. 3J), which also suggests the effects of *smn-1(gk)* are not just developmental in origin. In contrast, when we treated worms with neuronal-specific RNAi, we saw no effect of the gene knockdown on paralysis, and very little effect on neurodegeneration (Fig. 3K,L). Together, these data point to an important role for ER stress in non-neuronal cells in animals with *smn-1* mutations, and, from a therapeutic standpoint, targeting ER stress in non-neuronal cells may be effective and can complement neuronal-focused therapies.

### Enhanced protection from targeting the NMJ and ER stress response

**Fig. 4.**
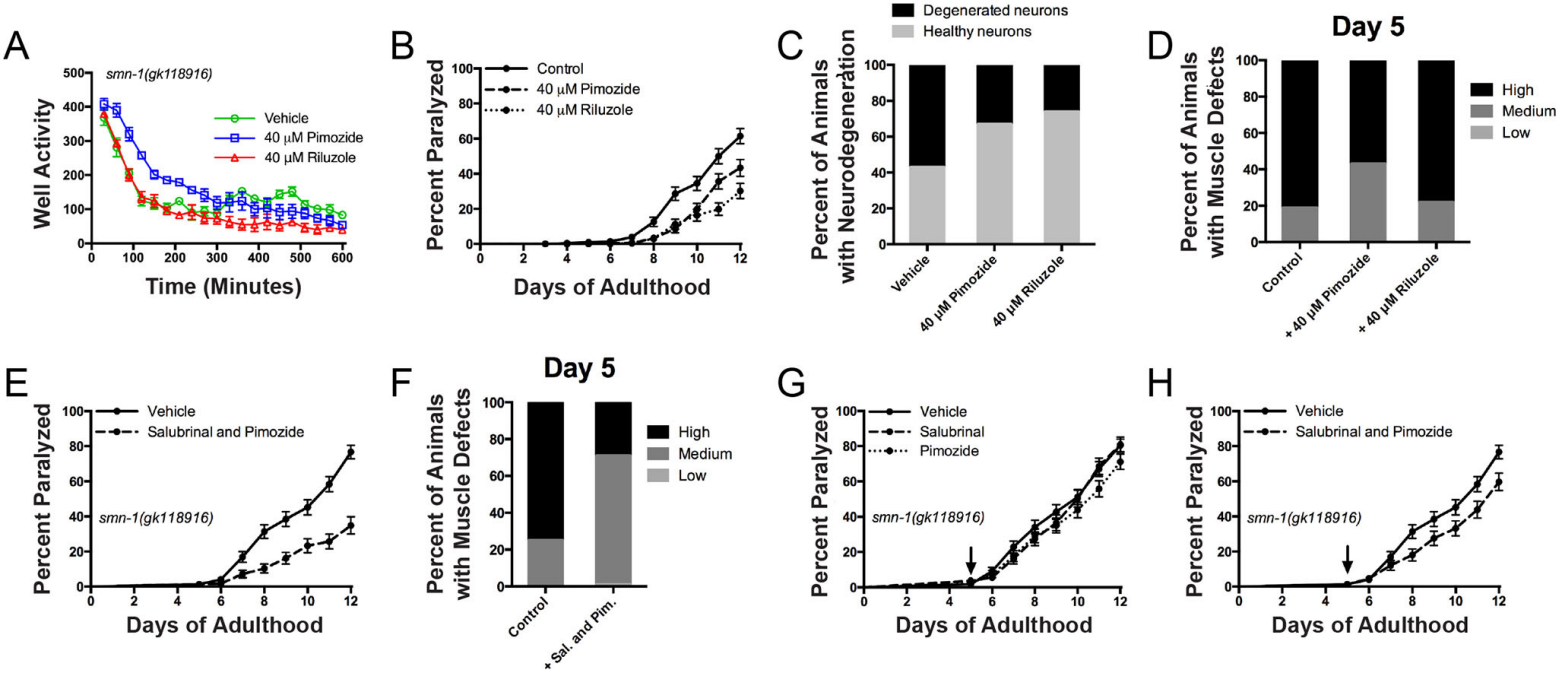
**Simultaneously targeting ER stress and NMJ stabilization has an additive effect on *smn-1(gk)* mutants.** (A) Treatment of *smn-1(gk)* mutant animals with 40 µM pimozide significantly improved the swimming behavior of the animals (*P*<0.0001), whereas 40 µM riluzole treatment significantly reduced it (*P*<0.0001). (B) Treatment with 40 µM pimozide or riluzole was able to significantly reduce paralysis in *smn-1(gk)* animals (*P*<0.001 and *P*<0.0001, respectively). (C) GABAergic motor neuron degeneration was significantly reduced in *smn-1(gk)* mutant animals upon pimozide or riluzole treatment (*P*<0.01 and *P*<0.001, respectively). (D) Muscle dysfunction was partially restored in *smn-1(gk)* animals upon treatment with pimozide (*P*<0.0001), but riluzole had no effect. (E) Treatment of *smn-1(gk)* animals simultaneously with 20 µM pimozide and 25 µM salubrinal effectively restored paralysis (*P*<0.0001). (F) Simultaneous treatment with both salubrinal and pimozide greatly restored muscle defects in *smn-1(gk)* mutants (*P*<0.0001). (G) Individually, treatment of *smn-1(gk)* mutants with salubrinal and pimozide from day 5 had no effect on paralysis. (H) Treatment of *smn-1(gk)* with both pimozide and salubrinal from day 5 resulted in a slight decrease of paralysis levels (*P*<0.01). Levels of morphological distortion were characterized as ‘low', ‘medium' or ‘high’ depending on the extent of GFP::MYO-3 disorganization in the cells. Data are mean±s.d. Statistical significance was determined using two-way ANOVA (A,D,F), a Mantel–Cox test (B,E,G) and a two-tailed unpaired Student's *t*-test (C).

Given the similarities between ALS and SMA, we decided to examine whether clinically-tested drugs for ALS could offer protective effects in a genetic model for SMA. We selected riluzole, a widely used ALS therapeutic, and pimozide, a neuroleptic recently identified by our group and validated in a small-scale clinical trial for ALS (Patten et al., 2017). We tested these two compounds for their ability to restore swimming behavior in *smn-1* mutants at day 5 of adulthood, and found that pimozide, but not riluzole, was able to significantly improve this behavior (Fig. 4A). However, both these drugs were able to suppress paralysis and neurodegeneration for *smn-1* mutants grown on solid medium (Fig. 4B,C). We next examined muscle cell defects, and we observed that of the two compounds, only pimozide suppressed muscle phenotypes in *smn-1* mutants (Fig. 4D), which is consistent with the hypothesis that pimozide acts by stabilizing the NMJ.

As we had identified compounds that could independently restore paralysis, neurodegeneration and muscle defects in *smn-1(gk)* animals, we decided to test whether they could act synergistically to better improve the health of the animals. We tested each compound at half the dose previously tested, or 20 µM for pimozide and 25 µM for salubrinal. We observed that this combination suppressed paralysis in *smn-1(gk)* animals, and rescued muscle morphology defects in *smn-1* mutants at day 5 of adulthood (Fig. 4E,F). However, these compounds, tested individually and applied from day 5 of adulthood, had little effect on suppressing paralysis in *smn-1* mutants (Fig. 4G); however, when both drugs were combined, and applied from day 5 of adulthood there was a reduction in paralysis (Fig. 4H). Together, our results suggest that early treatment by combining salubrinal and pimozide to simultaneously block ER stress and stabilize the NMJ is effective in rescuing *smn-1* mutant phenotypes. Therefore, this combination, if validated, could open up new therapeutic treatments for SMA patients. Furthermore, our results suggest that the timing window for delivery of this combination is limited and should be taken into consideration.

## DISCUSSION

In this study, we showed that a point mutation in *C. elegans smn-1* resulted in phenotypes of moderate severity, which allowed us to study the molecular signature of *smn-1*, shedding new light on SMA pathology. We showed that the onset of muscular dysfunction precedes neurodegeneration and that targeting therapies to muscle cells is more effective than neuronal delivery. The information gained from this model could not have been obtained from the extensively studied *C. elegans smn-1(ok355)* deletion mutant as this has a severe lethal phenotype, which masks the appearance of certain phenotypes or makes them difficult or impossible to study. For example, our model is the first nematode *smn-1* model with a genetic mutation to show neurodegeneration, a phenotype which could not be seen in the *ok355* mutant (Briese et al., 2009; Burt et al., 2006; Gallotta et al., 2016). By using an allele that generates mild SMA-like characteristics, much like the previously studied *smn-1(cb131)* (Sleigh et al., 2011), we were able to gradually monitor the onset of specific aspects of SMA pathology. However, despite the differences between our model and the reference *ok355* allele, many of the key phenotypes are conserved, including decreased lifespan, motility defects and changes in aldicarb sensitivity (Dimitriadi et al., 2016; Briese et al., 2009).

One of the key pieces of information gained in this study was the onset of muscle dysfunction in advance of neurodegeneration, and that modulating ER stress in non-neuronal cells was sufficient to rescue neurodegeneration. We hypothesize that muscle dysfunction may cause neurodegeneration in SMA whereby, after the onset of muscular dysfunction, it is possible that neurons could lose their NMJ connections and in turn degenerate. If verified in other systems, this information could change how we approach gene therapy for SMA. Current approaches focus on driving full-length *SMN1* into neuronal cells thereby re-expressing the full-length functional protein in these cells. To date, this approach has been very successful; however, the efficiency falls if it is delivered to SMA patients after 6 months of age, and we do not yet know the long-term effects of this approach. If our hypothesis is confirmed, the current success of neuronal gene therapy approaches could be attributed to SMN1-expressing neurons supporting muscles and slowing their degeneration. As we do not yet know the long-term clinical outcomes, we cannot determine whether the neuronal SMA gene therapy will continually support muscles, or whether there will come a point at which the neuronal support will no longer be sufficient and muscles will continue to degenerate. Furthermore, if our hypothesis is correct, it could help to explain why the current approach fails in patients after 6 months of age; it is possible muscle degeneration reaches a certain threshold beyond which the SMN1-expressing neuronal support fails to have any effect. Although it may take years before the necessary clinical information becomes known, our results suggest that muscle degeneration in SMA may be an important driver of neurodegeneration, and this information should be considered for future gene therapy efforts.

Although a combination of neuronal- and muscle-driven gene therapy for SMA will very likely benefit a large number of patients, it is also likely that not all affected individuals will respond equally to this therapeutic approach. Therefore, developing other therapeutic approaches using small molecules is also of utmost importance. In this study, we showed that a combination of two pharmacological compounds, pimozide and salubrinal, was more beneficial than each one individually. To target the ER stress in SMA, we used salubrinal, a specific inhibitor of eIF2a (Boyce et al., 2005). This showed promise in restoring paralysis, neurodegeneration and muscle defects in our *smn-1(gk)* model. The second compound, pimozide, is a neuroleptic that has been used as an anti-psychotic agent for many years, and was recently identified by our group in a chemical-genetic screen for TDP-43 toxicity in ALS, and was validated in a small phase IIa clinical trial (ClinicalTrials.gov Identifier: NCT02463825; Patten et al., 2017). In ALS, pimozide was hypothesized to stabilize the NMJ in ALS and, therefore, delayed the onset of neurodegeneration. Given the large clinical overlap between SMA and ALS, we decided to test pimozide in our SMA model, and it was also able to rescue key phenotypes. As pimozide was able to prevent muscle dysfunction, we believe this supports the hypothesis of pimozide stabilizing the NMJ. Interestingly, we also tested riluzole, an approved therapeutic for ALS, and although it was able to restore motility and neurodegeneration in *smn-1* mutants, it showed no effect on muscle health. Previously, riluzole was shown to activate calcium-activated potassium channels, which are mainly expressed in neurons in *C. elegans* (Dimitriadi et al., 2013). The differences between the effects of pimozide and riluzole in our *smn-1* model suggests a different mechanism of action and broadens the potential for pimozide to treat a range of neuromuscular disorders. The therapeutic application of pimozide resembles the stabilizing action that neuron-driven SMN1 expression has on muscles, and, therefore, could explain its beneficial effects. However, in combination, these drugs appear to act synergistically to target ER stress in non-neuronal cells and to stabilize the NMJ, further supporting muscle integrity.

Altogether, our data show an essential non-neuronal contribution of *smn-1* to neuronal survival. We also showed that a combination of therapies modulating ER stress and NMJ stabilization result in dramatically improved muscle health. We suspect the ER-modulating drugs are acting on muscle cells, the most likely cell type to respond to this treatment in the face of *smn-1* mutations. Although we acknowledge that these results need to be validated in other model systems, we propose that the information gained here could be applied to the development of future therapeutic efforts for SMA. Also, despite recent advances in gene therapy technology, its future remains uncertain and its success cannot be guaranteed in all patients. Therefore, it remains important to develop alternative small molecule therapeutics that can be orally administered to patients while they await a genetic diagnosis, or can be used in conjunction with current gene therapy approaches. It will be more advantageous if these molecules can be repurposed from other diseases, thereby eliminating the need for the time-intensive regulatory process required to take new molecules to market.

## MATERIALS AND METHODS

### *C. elegans* strains

All nematodes were cultured and handled as per standard methods. The following strains were used (strain name/genotype): N2, *smn-1(gk118916)*; LX929/*vsIs48[unc-17::GFP]*, *ufIs34[Punc-47::mCherry]*; DM8005/*raIs5[myo-3p::GFP::myo-3+rol-6(su1006)]*, *smn-1(gk118916); raIs5 [myo-3p::GFP::myo-3+rol-6(su1006)]*; TU3311/*uIs60 [unc-119p::YFP+unc-119p::sid-1]*, *smn-1(gk118916); uIs60[unc-119p::YFP+unc-119p::sid-1]*; VP303/*rde-1(ne219)*, *kbIs7 [nhx-2p::rde-1+rol-6(su1006)]*; NR350/*rde-1(ne219)*; *kzIs20 [hlh-1p::rde-1+sur-5p::NLS::GFP]*; and PS6192/*syIs243 [myo-3p::TOM20::mRFP+unc-119(+)+pBS Sk+].* Mutant *smn-*1 worms were outcrossed six times to wild-type N2 worms before use. Genotyping of the *smn-1(gk)* mutation was performed by high-resolution melting (HRM) using HRM MeltDoctor reagents (Applied Biosystems), and analyzed on HRM software (Applied Biosystems). Verification by Sanger sequencing was performed by Genome Quebec (McGill University). The *C. elegans smn-1 gk118916* allele was recapitulated in wild-type N2 animals by SunyBiotech, using CRISPR-Cas9; this strain was called *smn-1(syb1923)*. All experiments were carried out at 20°C and were repeated a minimum of three times.

### Paralysis assays

For all strains, 25 to 30 L4 animals were picked and placed on nematode growth medium (NGM) plates and scored daily, starting the following day at day 1 of adulthood. Worms were counted as paralyzed if they failed to move their body upon prodding with a platinum wire. Worms were considered dead if they failed to move their head when prodded and showed no pharyngeal pumping; dead worms were censored from statistical analyses. For paralysis assays with chemical compounds, worms were grown on standard NGM plates and transferred onto NGM+drug or NGM+DMSO control plates at the L4 stage. Worms were scored as described above. Together, a minimum of 250 animals were scored per genotype and condition.

### Lifespan assays

For all strains, 25 to 30 L4 animals were picked onto NGM plates and scored every second day from day 1 of adulthood until death. Worms were considered dead if they failed to respond to mechanical or heat stimulus, and showed no pharyngeal pumping. A minimum of 250 animals were scored per genotype.

### Aldicarb and levamisole assays

Worms were grown on NGM plates and transferred to NGM plates spiked with 1 mM aldicarb or 0.5 mM levamisole, at day 1 of adulthood. Paralysis was scored every 30 min for 2 h for aldicarb assays, or every 15 min for 1 h for levamisole assays. Animals were considered paralyzed if they failed to respond to gentle prodding with a platinum pick. A minimum of 250 animals were scored per genotype.

### Liquid culture motility assays

Synchronized age-matched worms were rapidly picked into 96-well plates containing M9 buffer. Thirty worms per genotype were picked into three wells each at either day 1 or day 5 of adulthood. Worm motility was quantified using a PhylumTech WMicrotracker-One over a period of 10 h.

### Gene expression assays

Synchronized age-matched animals were collected in M9 buffer at day 1 of adulthood. Animals were washed with M9 buffer four times to remove excess bacteria, and the supernatant was removed after the last wash step. Worms were then frozen at −80°C in 500 µl TRIzol Reagent (Thermo Fisher Scientific). After thawing, worms were homogenized using a 27 gauge ½ inch needle with a syringe, and another 500 µl of TRIzol was added. Samples were incubated at room temperature for 5 min before adding 200 µl of chloroform and letting them sit for an additional 2 min. Samples were then centrifuged at 12,000 ***g*** for 15 min to separate the phases. The aqueous phase was collected, and extraction was completed using an RNeasy Mini Kit (Qiagen) and its standard protocol. cDNA was synthesized using a SuperScript VILO cDNA Synthesis Kit (Invitrogen), and real-time PCR was performed, using TaqMan probes and standard TaqMan reagents, to quantify *smn-1* transcript levels (probes and reagents were purchased from Applied Biosystems). *Cdc-42.1* was chosen as the housekeeping gene. Gene expression assays were run on a QuantStudio 7 Flex instrument (Applied Biosystems) and data analysis was conducted using QuantStudio Real-Time PCR software.

### Microscopy experiments

All assays were carried out using a Zeiss Axio Observer inverted microscope. For neurodegeneration assays, worms were immobilized in 5 mM levamisole and mounted on slides with 2% agarose pads. Scoring of axonal breaks in either GABAergic or cholinergic motor neurons was performed *in vivo* with day 9 animals. For neurodegeneration assays with drugs, worms were grown on NGM plates until the L4 stage and transferred to NGM+drug or NGM+DMSO control plates, and maintained up to day 9 for imaging. A total of 100 animals were scored per genotype or treatment condition. For muscle degeneration assays, worms were immobilized with 0.05% sodium azide and imaged at day 3 or 5 of adulthood. Animals were scored as having either low, medium or high levels of morphological defects. In total, 100 animals were scored. For scoring mitochondrial morphology, age-synchronized day 1 and day 5 adults were immobilized in 5 mM levamisole dissolved in M9, and mounted on slides with 2% agarose pads. The mitochondria were scored as linear, intermediate or fragmented. A total of 100 worms were scored per condition.
